# Comprehensive Clinic-Pathological Characteristics of Cervical Cancer in Southwestern China and the Clinical Significance of Histological Type and Lymph Node Metastases in Young Patients

**DOI:** 10.1371/journal.pone.0075849

**Published:** 2013-10-09

**Authors:** LingYun Yang, XiBiao Jia, NingWei Li, Cen Chen, Yi Liu, HongJing Wang

**Affiliations:** Department of Gynecology and Obstetrics, West China Second University Hospital, Chengdu, Sichuan University, Sichuan, P.R.China; Johns Hopkins University, United States of America

## Abstract

**Objective:**

To analyze the clinic-pathological characteristics of women with cervical cancers in southwestern China and discuss the features and prognosis of young patients.

**Methods:**

A retrospective study was performed, which consisted of 1,543 patients diagnosed with cervical cancer and underwent treatment at West China Second University Hospital between November 2005 and December 2010. Among them, 154 young patients with surgical procedures between November 2005 and December 2008 were selected for a 5-year follow-up and prognostic analysis.

**Results:**

The proportion of advanced FIGO stage in patients aged over 35 years was higher than in patients aged 35 years or younger (55.1% vs 38.8%, *P*<0.001), and strong correlation was found between FIGO stages and the postoperative pathological risk factors (*P*<0.05). 312 patients (20.2%) were under 35 years old in the last 5 years. The proportion of cervical adenocarcinoma remained high in young patients (13.6%), and young women with adenocarcinoma had a higher rate of LN metastases, comparing with those with squamous cell carcinoma (42.9% vs 15.8%, *P* = 0.004). Young patients with adenocarcinoma had shorter progression-free survival than those who had squamous cell carcinoma (*P* = 0.024). Patients aged 35 years or younger with positive postoperative pathological risk factors had shorter progression-free survival, comparing with those with negative factors (*P*<0.01).

**Conclusion:**

Patients over 35 years were preliminarily diagnosed as advanced FIGO stage and they were more likely to have deep stromal invasion, LVSI, LN metastases, parametrial and surgical margin involvement. Regarding to young patients, cervical adenocarcinoma increased the risk of LN metastases and positive postoperative pathological risk factors could apparently worsen the prognosis. Histological type and LN metastases were independent prognostic factors for young patients in southwestern China. We re-emphasize the importance of health education and regular smear screening for elder women, and more attention should be paid to young patients with adenocarcinoma or LN metastases.

## Introduction

Carcinoma of the uterine cervix ranks as the second malignant cancer and the third leading cause of cancer deaths in women. Approximately 370,000 new cases are diagnosed annually with a 50% death rate [1], [2]. Population-based cervical cancer screening and advanced medical treatments have reduced the incidence by 40%–50% and the mortality by 60% in many developed countries. However, the morbidity of cervical cancer in China is still high with almost one-third of the total number worldwide, which remains one of the most important issues in women's health care [3]. In southwestern China, especially in some rural undeveloped regions, the problem is particularly severe due to the deficiency of regular screening and health care facilities. Previous studies have revealed that ages, FIGO stage, histologic types, depth of stromal invasion (DSI), lymph vascular space invasion (LVSI), and lymph nodes (LN) metastases are aggressive characteristics of cervical cancer, which are regarded as significant factors for treatment, prognosis and recurrence [4]–[7]. Somehow, these crucial clinic-pathological features have not previously been systematically analyzed and summarized in the last 5 years in southwestern China.

According to some epidemiological reports, the incidence of cervical cancer in young women is rising [8]–[11], while adenocarcinoma is more likely to be found among young patients [12], [13]. Several authors have reported that carcinoma of cervix had a poorer prognosis and survival rates in young patients than in elder women [10], [14]–[17], while others found that age has no relevance with the prognosis [18]–[21]. This remained a controversial issue. Additionally, considering the quality of life (QOL), a primary surgery with the radical hysterectomy without bilateral salpingo-oophorectomy is usually performed for young women with cervical cancer [22], and patients with postoperative pathological risk factors, such as bulky tumor size, DSI≥1/2, LVSI, LN metastases, parametrial and surgical margin involvement, should often receive adjuvant radiotherapy or concurrent chemotherapy, according to the National Comprehensive Cancer Network (NCCN) clinical practice guidelines of cervical cancer. However, to our best knowledge, there were no systematic studies that compared and analyzed the clinical significance of postoperative pathological risk factors for young women with cervical cancer in southwestern China by assessing the relevance to survivals.

Consequently, the aim of this study was to analyze the clinic-pathological characteristics of women with cervical cancers in southwestern China. The ages, FIGO stages, histology, and the postoperative pathological risk factors were examined. We also discussed the features and prognosis of young patients who were under 35 years old.

### Patients and Methods

This study was performed at the department of gynecology and obstetrics of West China Second University Hospital, Sichuan, China.

First, a retrospective study was performed. It consisted of 1,543 eligible patients who had been diagnosed with cervical cancer and were undergoing treatment at our hospital between November 2005 and December 2010. Patients were divided into 2 study groups: aged 35 years or younger and over 35 years. Surgical procedures and treatment were selected according to the NCCN clinical practice guidelines of cervical cancer. Clinical and pathological characteristics of the study groups were examined. Correlation analysis and multivariate analysis were employed to further calculate the relevance of FIGO stages to ages and the postoperative pathological risk factors.

Second, in order to discuss the characteristics and prognosis of young patients, 154 young patients with surgical procedures between November 2005 and December 2008 were selected for 5-year follow-up and prognostic analysis. The follow-up included interval history, gynecological examination and cervical smear testing in every 3 months for 1 year, every 6 months for another 2 years, and then annually. Adjuvant radiotherapy or concurrent chemotherapy was performed for patients with postoperative pathological risk factors. Because no invasive examination and sample collection was involved, only verbal consents were required and obtained from young patients or the parents of the patients who were under 18 years old. All verbal consents were documented and listed in a table. The follow-up and consent procedures were approved by Sichuan University Medical Ethical Committee.

Statistical analysis was performed using χ^2^ test, fisher exact test, pearson correlation coefficient and logistic regression analysis. Overall survival was obtained and evaluated by the Kaplan-Meier curve and log-rank test. Prognostic factors were analyzed using Cox proportional hazard models by stepwise selection. *P*<0.05 was regarded as statistical significance. The SPSS program version 16.0.2 was used for statistical analysis.

## Results

A total of 312 patients (20.2%) were 35 years or younger between November 2005 and December 2010. Table 1 summarized the clinic-pathological characteristics of the study groups. The proportion of being in the advanced FIGO stage (stage IIA-IV) in patients aged over 35 years was higher than the proportion in patients aged 35 years or younger (55.1% vs 38.8%, *P*<0.001). Patients aged 35 years or younger had a higher rate of bulk tumor size, comparing with patients over 35 years (30.8% vs 23.3%, *P* = 0.008). However, no significant difference of tumor differentiation was found between the two groups.

**Table 1:** Clinic-pathological characteristics of the study groups.

	N	≤35 years	>35 years	P
		(n = 312, 20.2%)	(n = 1231, 79.8%)	
Age, year				
Median		33.0	44.0	
Range		17–35	36–75	
Stage				<0.001
IA1	133 (8.6)	40 (12.8)	93 (7.6)	
IA2	52 (3.4)	17 (5.4)	35 (2.8)	
IB1	393 (25.5)	97 (31.1)	296 (24.0)	
IB2	165 (10.7)	37 (11.9)	128 (10.4)	
IIA	368 (23.8)	53 (17.0)	315 (25.6)	
IIB	411 (26.6)	64 (20.5)	347 (28.2)	
III	18 (1.2)	4 (1.3)	14 (1.1)	
IV	3 (0.2)	0 (0.0)	3 (0.2)	
Differentiation				1.000
G1/G2	185 (12.0)	37 (11.9)	148 (12.0)	
G3	1358 (88.0)	275 (88.1)	1083 (88.0)	
Size				0.008
<4cm	1160 (75.2)	216 (69.2)	944 (76.7)	
≥4cm	383 (24.8)	96 (30.8)	287 (23.3)	
Histological type				0.574
Squamous	1325 (85.9)	265 (84.9)	1060 (86.1)	
Non-squamous	187 (12.1)	41 (13.1)	146 (11.9)	
Adenocarcinoma	78 (5.1)	18 (5.8)	60 (4.9)	
Adenosquamous cell	95 (6.2)	20 (6.4)	75 (6.1)	
MMMT	2 (0.1)	1 (0.3)	1 (0.1)	
Small cell	16 (1.0)	5 (1.6)	11 (0.9)	
Clear cell	6 (0.4)	0 (0.0)	6 (0.5)	
Leiomyosarcoma	2 (0.1)	1 (0.3)	1 (0.1)	
Adenosarcoma	2 (0.1)	0 (0.0)	2 (0.2)	
Others	17 (1.1)	2 (0.6)	15(1.2)	
DSI				0.014
<1/2	622 (40.3)	145 (46.5)	477 (38.7)	
≥1/2	921 (59.7)	167 (53.5)	754 (61.3)	
LVSI				0.397
Negative	951 (61.6)	199 (63.8)	752 (61.1)	
Positive	592 (38.4)	113 (36.2)	479 (38.9)	
LN metastases				0.299
Negative	1243 (80.6)	258 (82.7)	985 (80.0)	
Positive	300 (19.4)	54 (17.3)	246 (20.0)	
Parametrial involvement				0.367
Negative	1443 (93.5)	288 (92.3)	1155 (93.8)	
Positive	100 (6.5)	24 (7.7)	76 (6.2)	
Surgical margin involvement				1.000
Negative	1496 (97.0)	303 (97.1)	1193 (96.9)	
Positive	47 (3.0)	9 (2.9)	38 (3.1)	

As shown in Table 1, non-squamous histological type included adenocarcinoma, adenosquamous cell carcinoma, malignant mixed mullerian tumor (MMMT), small cell carcinoma, clear cell carcinoma, leiomyosarcoma, adenosarcoma and other classified carcinoma. Squamous cell carcinoma was the main histological type. However, there was no significant difference of the histological type between the two groups. For the postoperative pathological risk factors, a lower rate of DSI≥1/2 was found in patients aged 35 years or younger, comparing with the rate in patients aged over 35 years (53.5% vs 61.3%, *P* = 0.014). In addition, the differences in LVSI, LN metastases, parametrial and surgical margin involvement between the two groups were insignificant.

A positive correlation was found between FIGO stages and the postoperative pathological risk factors (*P*<0.05) (Table 2). Furthermore, multivariate analysis revealed that ages, DSI, LN metastases and parametrial involvement were significantly related to FIGO stages (*P*<0.05) (Table 3).

**Table 2:** The correlation between FIGO stage and postoperative pathological risk factors.

	Early stage	Advanced stage	r	P
	(n = 1111, 72.0%)	(n = 432, 28.0%)		
DSI			0.280	0.000
<1/2	543 (48.9)	79 (18.3)		
≥1/2	568 (51.1)	353 (81.7)		
LVSI			0.143	0.000
Negative	733 (66.0)	218 (50.5)		
Positive	378 (34.0)	214 (49.5)		
LN metastases			0.201	0.000
Negative	950 (85.5)	293 (67.8)		
Positive	161 (14.5)	139 (32.2)		
Parametrial involvement			0.164	0.000
Negative	1067 (96.0)	376 (87.0)		
Positive	44 (4.0)	56 (13.0)		
Surgical margin involvement			0.066	0.010
Negative	1085 (97.7)	411 (95.1)		
Positive	26 (2.3)	21 (4.9)		

**Table 3:** The relevance of FIGO stage to age and postoperative pathological risk factors.

	OR	95%CI	P
Age	1.428	1.045∼1.950	0.025
DSI	3.502	2.647∼4.634	0.000
LVSI	1.193	0.931∼1.527	0.163
LN metastases	1.792	1.345∼2.387	0.000
Parametrial involvement	1.881	1.209∼2.924	0.000
Surgical margin involvement	1.162	0.614∼2.199	0.644

Between November 2005 and December 2008, 154 young patients were treated at our department. The clinic-pathological features were summarized in Table 4. Adenocarcinoma of cervix remained high in young patients (13.6%). A total of 136 patients (88.3%) were diagnosed with poor differentiation. Young patients with adenocarcinoma had a higher rate of well differentiation than those with squamous cell carcinoma (28.6% vs 9.0%, *P* = 0.010). There was no difference between patients with squamous cell carcinoma and patients with adenocarcinoma regarding their FIGO stages, treatment (treated with surgery or neoadjuvant chemotherapy), DSI, LVSI, parametrial and surgical margin involvement. However, young women with cervical adenocarcinoma had a higher rate of LN metastases than those with squamous cell carcinoma (42.9% vs 15.8%, *P* = 0.004).

**Table 4:** Clinic-pathological features of 154 patients aged 35 years or younger.

	N	Squamous	Adenocarcinoma	P
		(n = 133, 86.4%)	(n = 21, 13.6%)	
Stage				0.782
Early	106(68.8)	91(68.4)	15(71.4)	
Advanced	48(31.2)	15(31.6)	6(28.6)	
Differentiation				0.010
G1/G2	18(11.7)	12(9.0)	6(28.6)	
G3	136(88.3)	121(91.0)	15(71.4)	
Treatment				0.251
Surgery	123(79.9)	104(78.2)	19(90.5)	
Neoadjuvant chemotherapy	31(20.1)	29(21.8)	2(9.5)	
DSI				0.076
<1/2	64(41.6)	59(44.4)	5(23.8)	
≥1/2	90(58.4)	74(55.6)	16(76.2)	
LVSI				0.197
Negative	86(55.8)	77(57.9)	9(42.9)	
Positive	68(44.2)	56(42.1)	12(57.1)	
LN metastases				0.004
Negative	124(80.5)	112(84.2)	12(57.1)	
Positive	30(19.5)	21(15.8)	9(42.9)	
Parametrial and Surgical margin involvement				0.647
Negative	143(92.9)	124(93.2)	19(90.5)	
Positive	11(7.1)	9(6.8)	2(9.5)	

**Figure 1:**
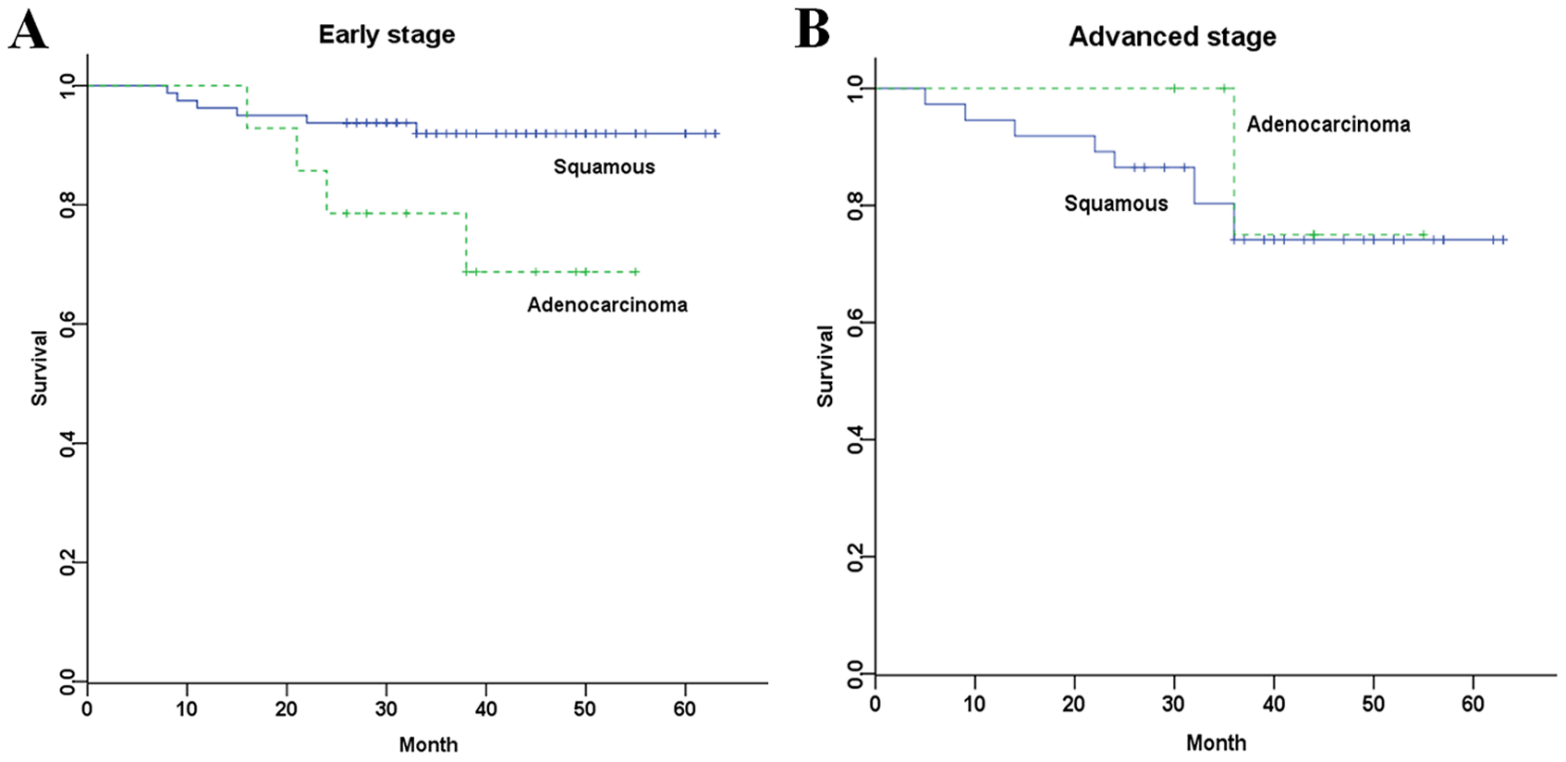
Kaplan-Meier analysis of progression-free survival. (A) PFS of young patients with early FIGO stage stratified by histological type (log-rank test *P* = 0.024). (B) PFS of young patients with advanced FIGO stage stratified by histological type (log-rank test *P* = 0.584).

Young patients in early FIGO stage who had adenocarcinoma had significantly shorter progression-free survival comparing with those who had squamous cell carcinoma (*P* = 0.024) (Fig. 1-A). However, no matter what the histological type is, young patients in advanced FIGO stage all had short progression-free survival (Fig. 1-B).

**Figure 2:**
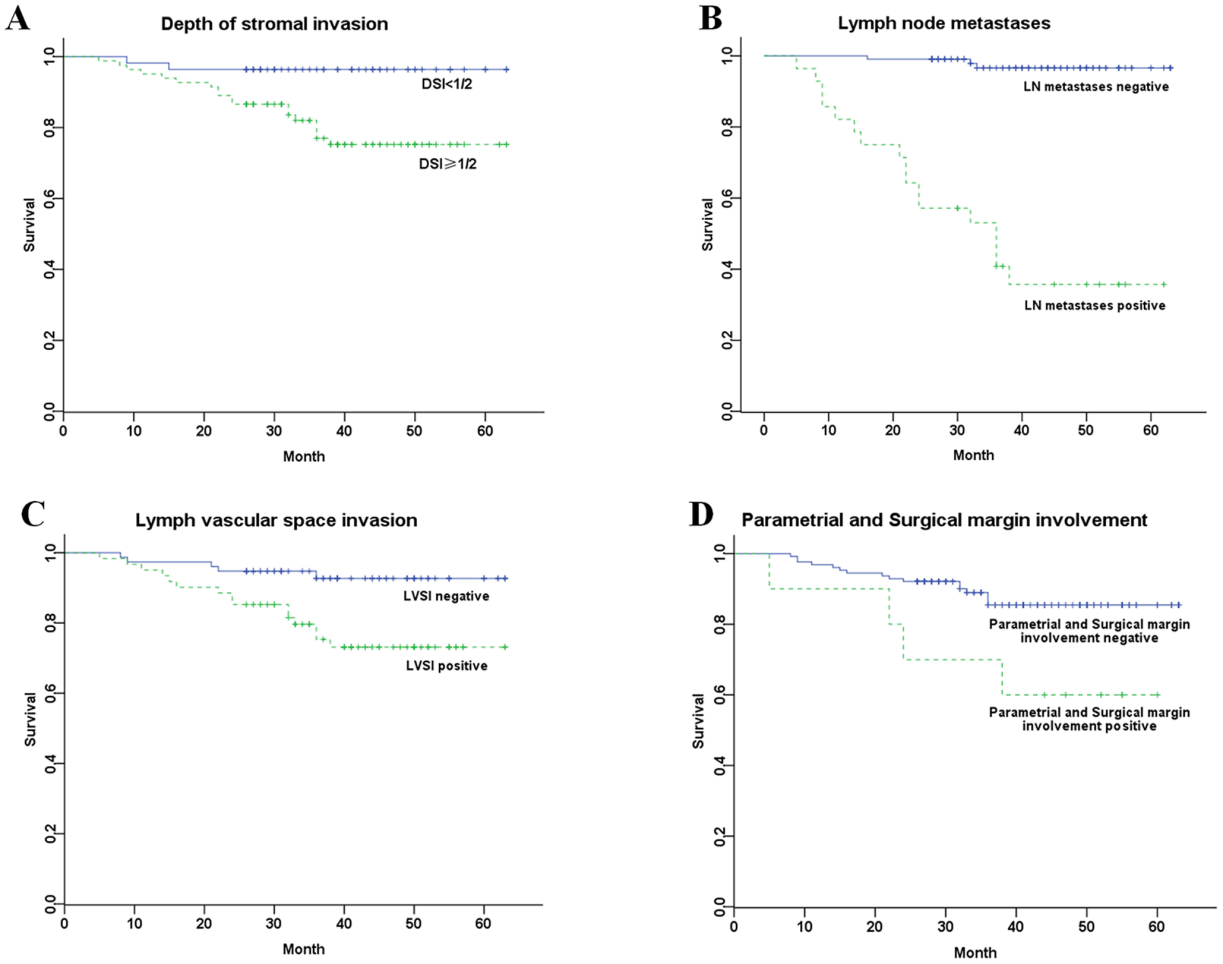
Progression-free survival of young patients with different pathological risk factors. (A) PFS of young patients stratified by DSI (log-rank test *P* = 0.005). (B) PFS of young patients stratified by LN metastases (log-rank test *P* = 0.000). (C) PFS of young patients stratified by LVSI (log-rank test *P* = 0.000). (D) PFS of young patients stratified by parametrial and surgical margin involvement (log-rank test *P* = 0.006).

Patients aged 35 years or younger with positive postoperative pathological risk factors (DSI≥1/2, LN metastases, LVSI, parametrial and surgical margin involvement) had significantly shorter progression-free survival than those with negative factors (*P*<0.01) (Fig. 2)

Prognostic analysis with the Cox proportional hazard model demonstrated that the prognostic factors of histological types and LN metastases were statistically significant among young patients in southwestern China (Table 5).

**Table 5:** Prognostic factors analyzed by Cox proportional hazard models for young patients.

Prognostic factors	Hazard ratio	95%CI	P
Stage	0.435	0.117∼1.622	0.215
Histological type	0.187	0.035∼0.988	0.048
Differentiation	0.644	0.131∼3.173	0.589
Treatment	0.727	0.220∼2.407	0.602
DSI	2.537	0.486∼13.253	0.270
LN metastases	48.533	11.238∼209.603	0.000
LVSI	2.113	0.689∼6.483	0.191
Parametrial and Surgical margin involvement	2.560	0.607∼10.801	0.201

## Discussion

In recent years, the cervical cancer rate is decreasing in some developed countries, although incidence remains high among Asian women [23]–[25]. The global annual incidence of cervical cancer was 493,200 in 2002, and the yearly death rate was 273,500, which is the third most common cancer in women worldwide [8]. According to epidemiological reports, 78% of cases occurred in developing countries, where cervical cancer was the second most frequent cause of cancer death in women [26]. In China, the morbidity of cervical cancer is still high with almost one-third of the total number worldwide, which remains a major health problem for women in China [3]. The problem is particularly severe in southwestern China due to the less-than–ideal medical condition.

The clinical stage of cervical cancer is definitely important for primary treatment and prognosis. Our results showed that the staging distribution was significantly different between the two study groups. Patients aged over 35 years had a higher proportion of being in the advanced FIGO stage, comparing with patients aged 35 years or younger. Furthermore, the preoperatively-diagnosed advanced FIGO stage might predict a higher potential of having postoperative pathological risk factors. In southwestern China, especially some rural regions, middle-aged and elderly women have little awareness of cervical cancer or the knowledge of its detection method. Besides, it is reported that pap smear screening is of great importance for detecting cervical cancer in the absence of symptoms [27], [28]. Consequently, this is why most patients over 35 years were preliminarily diagnosed as being in the advanced FIGO stage with poor prognosis. As a result, we re-emphasize the importance of health education and regular smear screening for middle-aged and elderly women in southwestern China.

Currently, the staging of cervical cancer remains largely a clinical evaluation, although surgical staging is more accurate, but it cannot be easily implemented in low resource regions [29]–[31]. The FIGO system limits the imaging examination to chest radiography, intravenous pyelography (IVP) and barium enema. It cannot comprehensively and systematically evaluate the involvement of pelvic tissues and organs. Our result demonstrated that patients in the advanced FIGO stage were more likely to have deep stromal invasion, LVSI, LN metastases, and parametrial and surgical margin involvement. Furthermore, multivariate analysis revealed that ages, DSI, LN metastases and parametrial involvement had statistically strong correlation to FIGO stages. Therefore, radiographic methods such as CT, MRI and PET-CT should be applied in order to thoroughly evaluate the DSI, LN metastases and parametrial involvement for patients in the advanced FIGO stage. In addition, the medical costs of physical examination should be reconsidered and reduced by health organizations.

Some reports have shown that the incidence of cervical cancer in young women is rising [8]–[11]. In our study, 312 patients (20.2%) were 35 years or younger in the last 5 years. This proportion is still higher than epidemiological data from other regions or countries [14], [32]. Persistent human papillomavirus (HPV) infection and early age of onset coitus, which causes atypical hyperplasia in young women, are regarded as the most crucial factors contributing to the development of cervical cancer [33]–[36]. The primary HPV infection site is squamous-columnar (S-C) junction of the cervical epithelium, where cells have active proliferation. For young women, the S-C junction is exposed at the exocervix, where cells are more vulnerable to HPV infection if she has an early age of onset coitus. In addition, estrogen-excessive pregnant status leads to cervical ectropion and vulnerability. As a result, immunization against HPV, especially for young women, is expected to prevent specific HPV related cervical cancer [37], [38].

It is reported that adenocarcinoma of the cervix has increased over the last 3 decades accounting for about 20% of all cervical cancers and the survival of patients with adenocarcinoma has not changed significantly. Meanwhile adenocarcinoma has been more likely to be found among young women [12], [13]. Our results showed that adenocarcinoma of cervix remained at a high level in young patients. This is probably caused by defection of cervical cytologic screening methods for adenocarcinoma [25], [39], and that young patients with cervical adenocarcinoma had a higher risk of LN metastases. Therefore, a shorter progression-free survival was found in young patients with adenocarcinoma, especially those in the early FIGO stage. In addition, we also demonstrated that positive postoperative pathological factors, such as deep stromal invasion, LVSI, LN metastases, and parametrial and surgical margin involvement, could apparently worsen the prognosis of young patients with cervical cancer. Previous study showed that FIGO stages, histological and postoperative pathological factors were found to be prognostic factors of cervical cancer [2], [4]–[7]. In this study, with the help of statistical analysis, histological type and LN metastases were found to be the most significant independent prognostic factors for young patients in southwestern China. Consequently, we should pay more attention to young patients with adenocarcinoma or LN metastases and reconsider the use of adjuvant radiotherapy or concurrent chemotherapy to improve their prognosis, although this method is hard to be accepted by some young women considering side effects, menopausal syndrome, economical affordability and quality of life (QOL).

In conclusion, we found that patients over 35 years who were preliminarily diagnosed as being in the advanced FIGO stage were more likely to have deep stromal invasion, LVSI, LN metastases, parametrial and surgical margin involvement. Regarding young patients, adenocarcinoma of cervix remained high and increased the risk of LN metastases, and positive postoperative pathological factors could apparently worsen the prognosis. Furthermore, histological types and LN metastases were the most significant independent prognostic factors for young patients in southwestern China. We therefore re-emphasize the importance of health education and regular smear screening for middle-aged and elderly women. In addition, we should pay more attention to young patients with adenocarcinoma or LN metastases and reconsider the use of adjuvant radiotherapy or concurrent chemotherapy to improve their prognosis, although this may be hard to be accepted.

## References

[1] Jemal A, Siegel R, Xu J, Ward E (2010) Cancer statistics, 2010. CA Cancer J Clin 60: 277–300. 20610543 10.3322/caac.20073

[2] Vinh-Hung V, Bourgain C, Vlastos G, Cserni G, De Ridder M, et al (2007) Prognostic value of histopathology and trends in cervical cancer: a SEER population study. BMC Cancer 23: 164. 10.1186/1471-2407-7-164 PMC1994954 17718897

[3] Cao ZY (2010) China Obstetrics and Gynecology 2nd Edition. Beijing: People's Health Press. 2012 p.

[4] Touboul C, Uzan C, Mauguen A, Gouy S, Rey A, et al (2010) Prognostic factors and morbidities after completion surgery in patients undergoing initial chemo-radiation therapy for locally advanced cervical cancer. Oncologist 15: 405–415. 20332143 10.1634/theoncologist.2009-0295 PMC3227965

[5] Marchiolé P, Buénerd A, Benchaib M, Nezhat K, Dargent D, et al (2005) Clinical significance of lympho vascular space involvement and lymph node micrometastases in early-stage cervical cancer: a retrospective case-control surgico-pathological study. Gynecol Oncol 97: 725–726. 15943983 10.1016/j.ygyno.2005.01.004

[6] Ho CM, Chien TY, Huang SH, Wu CJ, Shih BY, et al (2004) Multivariate analysis of the prognostic factors and outcomes in early cervical cancer patients undergoing radical hysterectomy. Gynecol Oncol 93: 458–464. 15099962 10.1016/j.ygyno.2004.01.026

[7] Delaloye JF, Pampallona S, Coucke PA, De Grandi P (1996) Younger age as a bad prognostic factor in patients with carcinoma of the cervix. Eur J Obstet Gynecol Reprod Biol 64: 201–205. 8820003 10.1016/0301-2115(95)02290-2

[8] Parkin DM, Bray F, Ferlay J, Pisani P (2005) Global Cancer Statistics, 2002. CA Cancer J Clin 55: 74–108. 15761078 10.3322/canjclin.55.2.74

[9] Shi JF, Qiao YL, Smith JS, Dondog B, Bao YP, et al (2008) Epidemiology and Prevention of Human Papillomavirus and Cervical Cancer in China and Mongolia. Vaccine 26: M53–59. 18945414 10.1016/j.vaccine.2008.05.009

[10] Clark MA, Naahas W, Markert RJ, Dodson MG (1991) Cervical cancer: women aged 35 and younger compared to women aged 36 and older. Am J Clin Oncol 14: 352–356. 1862767

[11] Elliott PM, Tattersall MH, Coppleson M, Russell P, et al (1989) Changing character of cervical cancer in young women. BMJ 298: 288–290. 2493898 10.1136/bmj.298.6669.288 PMC1835622

[12] Bulk S, Visser O, Rozendaal L, Verheijen RH, Meijer CJ (2003) Incidence and survival rate of women with cervical cancer in the Greater Amsterdam area. Br J Cance 89: 834–839. 10.1038/sj.bjc.6601157 PMC2394479 12942114

[13] Grisaru D, Covens A, Chapman B, Shaw P, Colgan T, et al (2001) Does histology influence prognosis in patients with early-stage cervical carcinoma? Cancer 92: 2999–3004. 11753977 10.1002/1097-0142(20011215)92:12<2999::aid-cncr10145>3.0.co;2-1

[14] Lau HY, Juang CM, Chen YJ, Twu NF, Yen MS, et al (2009) Aggressive characteristics of cervical cancer in young women in Taiwan. Int J Gynaecol Obstet 107: 220–223. 19716131 10.1016/j.ijgo.2009.07.029

[15] Kodama S, Kanasawa K, Honna S, Tanaka K (1991) Age as a prognostic factor in patients with squamous cell carcinoma of the uterine cervix. Cancer 68: 2481–2485. 1933786 10.1002/1097-0142(19911201)68:11<2481::aid-cncr2820681127>3.0.co;2-k

[16] Rutledge FN, Mitchell MF, Munsell M, Bass S, McGuffee V, et al (1992) Youth as a prognostic factor in carcinoma of the cervix: a matched analysis. Gynecol Oncol 44: 123–130. 1544591 10.1016/0090-8258(92)90027-g

[17] Robertson D, Fedorkow DM, Stuart GC, McGregor SE, Duggan MA, et al (1993) Age as a prognostic variable in cervical squamous cell carcinoma. Eur J Gynaecol Oncol 14: 283–291. 8344321

[18] Junor EJ, Symonds RP, Whatson ER, Lamont DW (1989) Survival of younger cervical carcinoma patients treated by radical radiotherapy in the West of Scotland 1964–1984. Br J Obstet Gyneco 96: 522–528. 10.1111/j.1471-0528.1989.tb03250.x 2757979

[19] Spanos WJ Jr, King A, Keeney E, Wagner R, Slater JM (1989) Age as a prognostic factor in carcinoma of the cervix. Gynecol Oncol 35: 66–68. 2792904 10.1016/0090-8258(89)90013-9

[20] Meanwell CA, Kelly KA, Wilson S, Roginski C, Woodman C, et al (1988) Young age as a prognostic factor in cervical cancer: analysis of population based data from 10,022 cases. Br Med J (Clin Res Ed) 296: 386–391. 10.1136/bmj.296.6619.386 PMC2544972 3125911

[21] Carmichael JA, Clarke DH, Moher D, Ohlke ID, Karchmar EJ (1986) Cervical cancer in women aged 34 and younger. Am J Obstet Gynecol 154: 264–269. 3946514 10.1016/0002-9378(86)90652-6

[22] Piver S, Rutledge F, Smith JP (1974) Five classes of extended hysterectomy for women with cervical cancer. Obstet Gynecol 44: 265–272. 4417035

[23] Barnholtz-Sloan J, Patel N, Rollison D, Kortepeter K, MacKinnon J, et al (2009) Incidence trends of invasive cervical cancer in the United States by combined race and ethnicity. Cancer Causes Control 20: 1129–1138. 19253025 10.1007/s10552-009-9317-z

[24] Wang SS, Carreon JD, Gomez SL, Devesa SS (2010) Cervical cancer incidence among 6 asian ethnic groups in the United States, 1996 through 2004. Cancer 116: 949–956. 20029972 10.1002/cncr.24843 PMC5736370

[25] Sherman ME, Wang SS, Carreon J, Devesa SS (2005) Mortality trends for cervical squamous and adenocarcinoma in the United States. Relation to incidence and survival. Cancer 103: 1258–1264. 15693030 10.1002/cncr.20877

[26] Kamangar F, Dores GM, Anderson WF (2006) Patterns of cancer incidence, mortality, and prevalence across five continents: defining priorities to reduce cancer disparities in different geographic regions of the world. J Clin Oncol 24: 2137–2150. 16682732 10.1200/JCO.2005.05.2308

[27] McMullin JM, De Alba I, Chávez LR, Hubbell FA (2005) Influence of beliefs about cervical cancer etiology on Pap smear use among Latina immigrants. Ethn Health 10: 3–18. 15841584 10.1080/1355785052000323001

[28] Hoque ME (2013) Awareness of cervical cancer, Papanicolau's smear and its utilization among female, final year undergraduates in Durban, South Africa. J Cancer Res Ther 9: 25–28. 23575070 10.4103/0973-1482.110350

[29] FUTURE II Study Group (2007) Quadrivalent vaccine against human papillomavirus to prevent high-grade cervical lesions. N Engl J Med 356: 1915–1927. 17494925 10.1056/NEJMoa061741

[30] Ault KA (2007) Effect of prophylactic human papillomavirus L1 virus-like-particle vaccine on risk of cervical intraepithelial neoplasia grade 2, grade 3, and adenocarcinoma in situ: a combined analysis of four randomised clinical trials. Lancet 369: 1861–1868. 17544766 10.1016/S0140-6736(07)60852-6

[31] Pecorelli S, Zigliani L, Odicino F (2009) Revised FIGO staging for carcinoma of the cervix. Int J Gynaecol Obstet 105: 107–108. 19342051 10.1016/j.ijgo.2009.02.009

[32] Liu L, Sun H (2008) The clinicopathologic characters and prognosis of cervical cancer in 831 young women under 35 years old. China Oncology 18: 298–301.

[33] Moscicki AB (2010) Human papillomavirus disease and vaccines in adolescents. Adolesc Med State Art Rev 21: 347–363. 21047033 PMC3057670

[34] Adamopoulou M, Kalkani E, Charvalos E, Avgoustidis D, Haidopoulos D, et al (2009) Comparison of cytology, colposcopy, HPV typing and biomarker analysis in cervical neoplasia. Anticancer Res 29: 3401–3409. 19661364

[35] Masumoto N, Fujii T, Ishikawa M, Mukai M, Ono A, et al (2004) Dominant human papillomavirus 16 infection in cervical neoplasia in young Japanese women; study of 881 outpatients. Gynecol Oncol 94: 509–514. 15297196 10.1016/j.ygyno.2004.05.011

[36] Frega A, Stentella P, De Ioris A, Piazze JJ, Fambrini M, et al (2003) Young women, cervical intraepithelial neoplasia and human papillomavirus: risk factors for persistence and recurrence. Cancer Lett 196: 127–134. 12860270 10.1016/s0304-3835(03)00218-0

[37] Gold MA, Tian C, Whitney CW, Rose PG, Lanciano R (2008) Surgical versus radiographic determination of para-aortic lymph node metastases before chemoradiation for locally advanced cervical carcinoma: a Gynecologic Oncology Group Study. Cancer 112: 1954–1963. 18338811 10.1002/cncr.23400

[38] Moore DH (2008) Surgical staging and cervical cancer: after 30 years, have we reached a conclusion? Cance 112: 1874–1876. 10.1002/cncr.23386 18348308

[39] Sasieni P, Castanon A, Cuzick J (2009) Screening and adenocarcinoma of the cervix. Int J Cancer 125: 525–529. 19449379 10.1002/ijc.24410

